# Time Trend in Psychotropic Medication Use in Spain: A Nationwide Population-Based Study

**DOI:** 10.3390/ijerph13121177

**Published:** 2016-11-24

**Authors:** Pilar Carrasco-Garrido, Valentín Hernández-Barrera, Isabel Jiménez-Trujillo, Jesús Esteban-Hernández, Alejandro Álvaro-Meca, Ana López-de Andrés, José Luis DelBarrio-Fernández, Rodrigo Jiménez-García

**Affiliations:** Preventive Medicine and Public Health Teaching and Research Unit, Health Sciences Faculty, Rey Juan Carlos University, Alcorcón 28922, Spain; valentin.hernandez@urjc.es (V.H.-B.); isabel.jimenez@urjc.es (I.J.-T.); jesus.esteban@urjc.es (J.E.-H.); alejandro.alvaro@urjc.es (A.Á.-M.); ana.lopez@urjc.es (A.L.-d.A.); jose.delbarrio@urjc.es (J.L.D.-F.); rodrigo.jimenez@urjc.es (R.J.-G.)

**Keywords:** psychotropic drug, predictors, sex differences, health survey, national trends

## Abstract

*Background:* We performed an epidemiologic study to analyze nationwide time trends in adult psychotropic drug use over a period from 2006 to 2012, and to identify those factors associated with the likelihood of consumption of these drugs during the study period; *Methods:* Cross-sectional study on psychotropic medication in the Spanish adult population. We used secondary individualized data drawn from the 2006 and 2012 Spanish National Health Surveys (SNHS). The dependent variable was the use of psychotropic drugs in the previous two weeks. Independent variables included socio-demographic characteristics, comorbidity, lifestyles and healthcare resource utilization. Using logistic multivariate regression models, we analyzed the temporal evolution of psychotropic medication consumption between 2006 and 2012 in both sexes; *Results:* The prevalence of psychotropic drug use was significantly greater in women (18.14% vs. 8.08% in 2012 (*p* < 0.05). In Spanish women, the variables associated with a greater probability of psychotropic use were, age, unemployment (adjusted odds ratio (AOR), 1.60; 95% CI, 1.24–2.07), negative perception of health or taking non-psychotropic drugs. Among men, psychotropic use is associated with presence of chronic disease, negative perception of health (AOR, 3.27; 95% CI, 2.62–4.07 in 2012) or inactive status; *Conclusions:* Between 2006 and 2012, the probability of having taken psychotropic drugs increased by 16% among women. Unemployed women aged ≥45 years with a negative perception of their health constitute a clear risk profile in terms of psychotropic drug use. Inactive men who have a negative perception of their health are the group most likely to consume psychotropic drugs.

## 1. Introduction

Health is affected by a series of determinants including socioeconomic conditions, working and living conditions, and social gradient [1]. In recent years, research on inequality in healthcare shows that morbidity and mortality are higher in people with a lower socioeconomic level than in those with a higher socioeconomic level [2,3].

Several studies have examined the effect of socioeconomic factors on mental health [4,5,6,7]. If we take into account educational level, occupational status, and income, it seems reasonable to suppose that these same factors could play a role in the effects of pharmacologic and psychosocial interventions and lead to social inequality in the response to treatment of psychiatric diseases [8,9].

Patients diagnosed with mental health problems are often prescribed a psychotropic drug (anxiolytics, antidepressants, or hypnotics-sedatives) [10,11,12]. According to the results for Spain in the European Study of the Epidemiology of Mental Disorders (ESEMeD) [13], 16% of the Spanish population reported having taken at least one psychotropic drug during the previous year. However, recent research has revealed that a high proportion of individuals with mental health disorders have not received an appropriate diagnosis or treatment. In fact, the results of some of these studies, such as that performed by the National Comorbidity Survey Replication (NCS-R), show that 58.1% of people with a depressive disorder have not visited a health center to obtain treatment for their problem [14].

In Spain, psychotropic drugs are available only by prescription. The scientific literature provides sufficient evidence that the prescription of a psychotropic drug is affected by various clinical and non-clinical factors (e.g., patient characteristics such as age and sex [15] and socioeconomic factors such as profession and social class [12]). Considering that consumption may be affected by adverse socioeconomic contexts, it is relevant to determine whether there have been any changes in the rate of consumption of psychotropic drugs in Spain during the recent period of instability [5], in comparison with the time before this situation arose [13].

In this context, we performed an epidemiologic study to analyze nationwide time trends in adult psychotropic drug use over a period from 2006 to 2012, and to identify those factors associated with the likelihood of consumption of these drugs during the study period.

## 2. Materials and Methods

We used individualized secondary data retrieved from the 2006 and 2012 Spanish National Health Surveys (SNHS) to conduct a nationwide, cross-sectional study on psychotropic drug use in individuals aged ≥25 years in Spain. SNHS are interview-based surveys undertaken by the Ministry of Health and Consumer Affairs that target a large sample of the non-institutionalized population in Spain. The surveys use multistage cluster sampling, with proportional random selection of primary and secondary sampling units (towns and sections, respectively); the final units (individuals) are selected using random routes and sex- and age-based quotas. The 2006 survey included 29,478 adults of both sexes interviewed between June 2006 and June 2007; the 2012 survey included 20,007 persons interviewed from July 2011 to June 2012. Details of National Health Survey methodology are reported elsewhere [16,17]. The variables included in our study were selected on the basis of a series of identically worded questions asked in the two surveys used.

The study population comprised people of both sexes aged ≥25 years. The study was based on 46,661 surveys, as follows: 27,310 from SNHS 2006 and 19,351 from SNSH 2012. The dichotomous dependent variables chosen were the answers “yes” or “no” to the question “In the last two weeks have you taken the following medicines and were they prescribed for you by a doctor?” with two possible answers: (1) tranquilizers (anxiolytics) or sedatives (anxiolytics) or sleeping pills (anxiolytics), and (2) antidepressants. The independent variables are the primary sociodemographic characteristics of the population, namely, age, sex, occupational status, educational level, total monthly household income in euros, and social class. The lifestyle-related variables were currently smoking (yes/no), consumption of any alcoholic beverages in the two weeks prior to the survey (yes/no), leisure time physical activity in the last week (intense or moderate vs. none), and self-reported body mass index (categorized as <25, 25–30, and >30). In order to assess the use of healthcare resources, respondents were asked about visits to a primary care physician (medical consultation in the preceding four weeks (yes/no)) and whether they had taken any type of medication, excluding psychotropic drugs, in the last two weeks. Self-reported presence of any chronic disease and self-rated health status (excellent/very good vs. fair/bad/very bad) were analyzed as dichotomous variables.

### 2.1. Ethical Statements

In accordance with Spanish legislation, there is no need for Ethics Committee approval, since the database was obtained from the Spanish Ministry of Health, Social Services and Equality webpage, where it is publicly available. The data was provided to us and therefore analyzed anonymously.

### 2.2. Statistical Analysis

We calculated the prevalence of psychotropic drug use for each of the two surveys according to the study variables. Pearson’s χ^2^ test was used for the bivariate comparison of proportions, and statistical significance was set at a *p* value < 0.05 (two-tailed). To estimate the independent effect of each of the study variables on the consumption of psychotropic drugs, we also obtained the corresponding adjusted odds ratio (AOR) using multivariate logistic regression analysis. Two models were generated, one for women and another for men.

Finally, to assess the time trend in the consumption of psychotropic drugs over the period 2006–2012, we combined the respective databases of the two SNHS and calculated the crude and adjusted odds ratios (crude odds ratio (COR) and AOR) for having consumed the psychotropic drug in 2012 compared with 2006, for both sexes. For calculation of the adjusted OR, all variables that proved to be predictive in either of the SNHS were included in the multivariate logistic regression.

Estimates were made using the “svy” function (survey command) of the STATA program (STATA Corp, College Station, TX, USA), which enabled us to incorporate the sampling design and weights into all statistical calculations (descriptive, χ^2^, and logistic regression). Statistical significance was established as a two-tailed α < 0.05.

## 3. Results

Our results showed that 16.79% of adults aged ≥25 years and interviewed in 2006 and 15.58% in the year 2012 reported having taken a psychotropic drug during the two weeks preceding the survey. Table 1 shows the frequency of psychotropic drug use (anxiolytics and antidepressants) in both sexes during the study period. The frequency of anxiolytic use increased in both sexes in 2012.

Data on psychotropic drug use in both sexes according to sociodemographic characteristics, lifestyle, and health-related study variables are shown in Table 2.

The prevalence of psychotropic drug use was significantly greater in women (18.14% (17.30–19.02) vs. 8.08% (7.43–8.78)) in 2012 for all the categories of variable analyzed and during the years 2006 and 2012 (*p* < 0.05). Psychotropic drug use was significantly more frequent in individuals with no formal schooling, unemployed people, and inactive people, especially women. Similarly, in both surveys, men and women with a negative perception of their health took more psychotropic drugs than persons with a good perception of their health (37% vs. 1.9% in women and 20.2% vs. 3.9% in men for the year 2012; *p* < 0.05).

The multivariate logistic regression analysis (Table 3) revealed the independent effect of each of the study variables (adjusted for the others) on psychotropic drug use in our sample for both sexes and during the years analyzed.

Analysis of patterns of use among women revealed that the variables that were independently and significantly associated with a greater probability of psychotropic drug use were age (particularly in the 45–64-year-old group in 2012 (AOR, 1.89; 95% CI, 1.55–2.30)) and occupational status (people who were unemployed in the SNHS 2012 (AOR, 1.60; 95% CI, 1.24–2.07) and inactive in the SNHS 2006 (AOR, 1.19; 95% CI, 1.01–1.42 and the SNHS 2012 (AOR, 1.88; 95% CI, 1.51–2.33)). The variables with the highest values of association among women in both 2006 and 2012 were negative perception of health (SNHS 2006, AOR, 3.63 (95% CI, 3.15–4.18); SNHS 2012, AOR, 3.60 (95% CI, 3.12–4.17)) and taking non-psychotropic drugs.

As for Spanish men, the health profile analysis showed that the presence of chronic disease, medical consultation, and negative perception of health were all likewise significantly associated with more frequent psychotropic drug use (SNHS 2006, AOR, 3.84 (95% CI, 3.07–4.80); SNHS 2012, AOR, 3.27 (95% CI, 2.62–4.07)). In both surveys, we found that inactive men were more likely to use psychotropic drugs (SNHS 2006 AOR, 2.96; 95% CI, 2.15–4.00; SNHS 2012 AOR, 1.88; 95% CI, 2.02–4.00).

To assess the time trend in the consumption of psychotropic drugs over the period 2006–2012, and taking 2012 as reference for having a higher consumption, analysis of the trend in psychotropic drug consumption from 2006 to 2012 in Spanish women, yielded a COR of 0.91 (95% CI, 0.84–0.90). On controlling for possible confounding variables, this statistical significance was maintained (AOR, 1.16; 95% CI, 1.06–1.28), meaning that in 2012 psychotropic drug consumption in women was 16% greater than that reported in 2006. Changes in trends of psychotropic drug consumption among Spanish men during the study period were not statistically significant.

## 4. Discussion

Our results show that the prevalence of prescription psychotropic drug use by Spanish adults reached 16.79% in 2006 and 15.58% in 2012. These values are lower than the 33.6% reported in a recent study on psychotropic drug prescription patterns in primary health centers [10], although they are higher than those found in other developed countries, such as the 5.92% reported by Athanasopoulos et al. in Greece based on a survey carried out by the National Center for Social Research [18], the 7.2% reported in the PATH Through Life Study in Australians of the same age group as those we studied [8], and the 8.6% reported by Ilyas et al. [19] for prescription of psychotropic drugs in England.

During the six-year study period, psychotropic drug use in Spain decreased by 1.21%. Use of antidepressants in particular decreased in 2012, in contrast to the results obtained by various researchers from several countries who reported a significant increase in prescription of psychotropic drugs during the last decade [9,20,21,22,23]. However, use of anxiolytics increased to 24.3%, consistent with the results obtained in an ecological study based on Spanish pharmacy dispensing data [24] and with those of Tsimtsiou et al. [12], who determined predictors of prescription of anxiolytics and hypnotics by general practitioners in England based on data from the UK Quality and Outcomes Framework. This increase in the consumption of anxiolytics could be because this drug class is used to treat a wide variety of conditions, whereas antidepressants are indicated for specific conditions.

Despite a 1.21% general decrease in the consumption of psychotropic drugs in Spain during the six years of our study, mainly due to a decline in antidepressant consumption, we must bear in mind that we could be facing a situation of under-declaration of drug consumption. In fact, there are studies, including some from our own country [10], that indicate an increase in consumption in recent years.

Being asked about antidepressant use may still spark socially conditioned responses from some people in Spain due to the negative connotations attached to their consumption. On the other hand, anxiolytic drug consumption may be considered more acceptable and so people may be less reluctant to admit taking this kind of medication. We can only speculate whether anxiolytic and hypnotic drugs are sometimes prescribed in order to counteract some of the side effects of antidepressive drug consumption such as restlessness, anxiety, and problems sleeping. Anxiolytics and hypnotics present addictive properties, which complicate cessation of these drugs for long-term users. Long-term variations in the prevalence of anxiolytic and hypnotic use probably reflect changes of some prescriptions from routine to PRN (pro re nata), or vice versa.

However, it should be noted that when these drug consumption trends are analyzed by gender, we observe a 16% increase among Spanish women, but no changes among men. For decades, the higher prescription and consumption of psychotropic drugs among women has been explained by a greater frequency of affective disorders in women or by their greater vulnerability in our society. This could be due to an increased predisposition among women to recognize and express their symptomatology and to seek medical assistance more frequently.

Sex differences in prescription and use of medications are well documented in the literature, and several variables have been shown to affect prescribing decisions and the outcome of treatment [18,23]. Nevertheless, despite the large body of scientific evidence, the problem remains. In the case of psychotropic drugs, a common denominator in these studies was that women consumed more psychotropic drugs than men [21,22], regardless of whether the patients were young or elderly women [15] or of the degree of social deprivation [9,11].

Our data revealed that Spanish women consume more psychotropic drugs than men, a trend which increased by 16% between 2006 and 2012 (AOR, 1.16; 95% CI, 1.06–1.28). In socioeconomic terms, the typical patient taking psychotropic drugs is a woman aged ≥45 years. A significant association has been found between this profile and psychotropic drug use, consistent with findings from studies such as the Baltimore Epidemiologic Catchment Area (ECA) Study [25], which shows ≥45 years to be the age group with the highest prevalence of behavioral disorders in women, or the Swedish Women’s Health in Lund Area Study (WHILA) (Study [26], which was performed to determine the prevalence of psychotropic drug use in middle-aged women and to examine the association with various social factors and health profiles.

From an occupational viewpoint and consistent with the greater incidence of female unemployment in recent years, Spanish women who were unemployed or inactive in 2012 (AOR, 1.60; 95% CI, 1.24–2.07), were more likely to consume psychotropic drugs than women who were employed. However, in the case of men, we found that inactive men consumed almost three times more psychotropic drugs than those who were employed (AOR, 2.85; 95% CI, 2.02–4.00). Researchers point out that the mental health of people who are unemployed is poorer than that of people who are employed [6,7]. It has also been suggested that sex differences in the effect of unemployment on mental health are associated with differences in the positions and roles of men and women in society and family and that these roles are associated with differences in the psychosocial and financial need for employment [27].

Unemployed men and women may seek professional care for mental health problems more frequently than might be expected, as shown in studies that support the hypothesis of “medicalization of unemployment”, that is, the use of healthcare not only as a response to mental health problems, but also as a way of dealing with unemployment [28]. However, between 2006 and 2012, psychotropic drug use decreased from 9.9% to 5.8% in men and increased from 13.7% to 14.9% in women.

Similarly, we could argue that prescription of psychotropic drugs in our study also seems to be associated with the number of visits to the doctor in both men and women.

Our analysis of variables associated with comorbidity and health-related behavior revealed that chronic diseases and smoking were predictors of psychotropic drug use by Spanish women. Female smokers in Spain are more likely to use psychotropic drugs than non-smoking women. Certain drugs are commonly consumed by smokers, and there is a close relationship between tobacco smoking and psychiatric disorders (a higher proportion of individuals with mental health conditions smoke compared with the general population). We also found a negative association between consumption of alcoholic beverages and the use of psychotropic drugs in both men and women in the two surveys. This finding could be interpreted as appropriate adherence by the patient to the advice of and regimen recommended by the physician when a psychotropic drug is prescribed, although we must remember that affective disorders, especially in men, are very often hidden by behavioral alterations or alcoholism.

Of note, concomitant use of non-psychotropic drugs was one of the variables most closely associated with psychotropic drug use by women (AOR, 3.00; 95% CI, 2.48–3.64). This finding is consistent with data from the annual Health Survey for England 2013, which found that 24% of women reported having taken at least three prescription drugs during the year [29].

We found that a considerable percentage of men and women reported being in poor health and that there was a significant association between men and women with a negative perception of their health and consumption of prescription psychotropic drugs (up to three-fold greater probability of consuming psychotropic drugs than persons with a positive perception of their health). Our results agree with those of several authors in that they reveal negative perception of health to be a predictor of psychotropic drug use, as shown in the results of a recent study carried out in Finland, where psychotropic drug use was almost double among people who had a poorer perception of their health (AOR, 1.90; 95% CI, 1.01–3.68) [30].

The main strengths of our study are its use of a representative sample to assess the prevalence of psychotropic drug use in Spanish adults during the period 2006–2012 and our ability to control for a broad range of major covariates, including socioeconomic factors and health-related variables.

Our study is limited by the nature of national health surveys. First, as the SNHS has not been validated for drug use, it is difficult to generalize the conclusions drawn from the results. Second, national health surveys are based on self-reported data, with the result that the prevalence of psychotropic drug use may be underestimated. Third, some replies may be socially conditioned (owing to the sociocultural association between psychotropic drug use and mental health problems), thus potentially affecting the demand for prescriptions of these drugs. Fourth, the Spanish National Health Survey, rather than identifying specific active pharmaceutical ingredients, identifies groups of medicines for specific diseases, conditions or disorders. Lastly, given that the initial response rate to the SNHS 2006 was 65% and the initial response rate to the SNHS 2012 was 61%, the possibility of a non-response bias must be taken into consideration [16,17].

## 5. Conclusions

Between 2006 and 2012, the probability of having taken psychotropic drugs increased by 16% in Spanish women. Our results confirm that unemployed women aged >45 years with a negative perception of their health constitute a clear risk profile in terms of psychotropic drug use.

Unemployed men who have a negative perception of their health are the group most likely to consume psychotropic drugs. No increase in consumption was observed in this group during the study period.

The increase in psychotropic drug use by women could be associated with an increased frequency of anxiety disorders. While abundant data can be found on social and cultural causes and on prescription and suitability of treatment, the importance of work-related factors has not received sufficient attention.

## Figures and Tables

**Table 1:** Anxiolytics and antidepressant drug used in Spanish adult population. Spanish National Health Surveys (SNHS) 2006 and 2012 (Percentage and 95% Confidence Intervals).

Psychotropic Drugs	Female ^†^	Male ^ǂ^
Anxiolytics SNHS 2006	15.69 (14.98–16.42)	7.32 (6.71–7.98)
Anxiolytics SNHS 2012	16.79 (15.97–17.63)	7.51 (6.87–8.19)
Antidepressant SNHS 2006	8.89 (8.34–9.47)	3.58 (3.14–4.07)
Antidepressant SNHS 2012	7.47 (6.92–8.05)	1.82 (1.55–2.20)

**^†^** Statistically significant differences (*p* < 0.05) in women SNHS 2006–SNHS 2012; **^ǂ^** Statistically significant differences (*p* < 0.05) in men SNHS 2006–SNHS 2012.

**Table 2:** Prevalence of psychotropic drug use in adult residents in Spain according to sociodemographic variables, lifestyle; health profile and healthcare resources. Spanish National Health Surveys 2006 and 2012.

Variables	Categories	Women		Men	
		SNHS 2006 % (95% CI)	SNHS 2012 % (95% CI)	SNHS 2006 % (95% CI)	SNHS 2012 % (95% CI)
Age * ^+^ ** ^++^	25–44 ^†^	9.1 (8.2–10.1)	7.7 (6.7–8.8)	5.0 (4.2–5.9)	4.8 (3.9–5.8)
	45–64 ^†^	21.8 (20.4–23.3)	19.3 (17.8–20.8)	9.7 (8.5–11.1)	8.5 (7.4–9.7)
	65 or more	32.6 (30.8–34.4)	33.3 (31.5–35.3)	16.1 (14.4–17.9)	14.5 (12.9–16.3)
Nationality * ^+^ ** ^++^	Spanish	20.1 (19.3–20.9)	19.4 (18.5–20.3)	9.4 (8.7–10.2)	8.5 (7.8–9.2)
	Immigrants ^ǂ^	10.5 (8.2–13.4)	8.4 (6.1–11.4)	2.2 (1.3–3.8)	4.9 (2.9–8.0)
Marital status * ^+^ ** ^++^	Not Married	22.3 (20.8–23.7)	21.0 (19.6–22.4)	9.9 (8.5–11.4)	8.2 (7.1–9.5)
	Married ^†^	17.7 (16.8–18.7)	16.2 (15.2–17.3)	8.3 (7.5–9.1)	8.0 (7.2–8.9)
Occupational status * ^+^ ** ^++^	Employed	11.5 (10.5–12.6)	8.3 (7.4–9.4)	4.5 (3.9–5.2)	4.4 (3.8–5.2)
	Unemployed	13.7 (11.3–16.5)	14.9 (12.6–17.6)	9.9 (7.0–13.8)	5.8 (4.4–7.6)
	Inactive ^†^	25.9 (24.7–27.1)	28.2 (26.7–29.6)	18.1 (16.5–20.0)	16.4 (14.8–18.1)
Social class * ** ^++^	High	13.6 (12.1–15.3)	14.5 (12.7–16.5)	7.9 (6.6–9.5)	6.6 (5.4–8.1)
	Middle ^†^	19.1 (18.0–20.3)	16.9 (15.5–18.4)	9.1 (8.2–10.1)	7.1 (6.1–8.3)
	Low	21.9 (20.4–23.6)	19.9 (18.6–21.3)	8.3 (7.1–9.8)	9.2 (8.2–10.4)
Educational level * ^+^ ** ^++^	No formal education	33.3 (31.0–35.7)	31.7 (29.3–34.2)	14.1 (11.7–16.8)	13.6 (11.2–16.3)
	Junior school ^†^	23.7 (22.2–25.1)	27.8 (25.1–30.7)	9.6 (8.4–10.9)	13.1 (10.8–16.0)
	High school ^†^	13.3 (12.1–14.7)	16.3 (15.1–17.5)	8.1 (6.9–9.4)	7.5 (6.7–8.5)
	University	10.0 (8.76–11.4)	8.8 (7.5–10.3)	6.5 (5.4–7.8)	4.6 (3.7–5.7)
Monthly income * ^+^ ** ^++^	900 €	28.4 (26.7–30.2)	26.2 (24.4–28.0)	14.7 (12.9–16.7)	12.3 (10.6–14.1)
	901–1800 €	18.4 (17.1–19.6)	16.7 (15.1–18.5)	8.1 (7.2–9.3)	8.3 (7.1–9.8)
	more than 1800 €	11.2 (9.9–12.7)	12.2 (10.6–13.9)	6.1 (5.1–7.3)	5.3 (4.3–6.4)
Smoking habit * **	Non smoker	20.0 (19.1–21.0)	19.0 (18.0–20.0)	8.6 (7.9–9.5)	8.5 (7.7–9.4)
	Smoker	15.5 (14.0–17.1)	15.3 (13.5–17.2)	8.8 (7.6–10.1)	7.2 (6.1–8.4)
Alcohol consumption * ^+^ ** ^++^	No	21.7 (20.7–22.8)	21.4 (20.3–22.6)	13.5 (11.9–15.2)	12.1 (10.8–13.6)
	Yes ^†^	15.1 (14.0–16.3)	12.6 (11.5–13.9)	6.8 (6.1–7.5)	5.9 (5.2–6.7)
Physical activity * ** ^++^	Yes ^†^	18.1 (17.1–19.2)	14.9 (13.9–16.1)	8.3 (7.5–9.2)	6.6 (5.8–7.5)
	No	20.2 (19.0–21.4)	21.4 (20.1–22.7)	9.3 (8.2–10.5)	10.2 (9.1–11.4)
Body mass index Kg/sq.m * ^+^ ** ^++^	<25	13.9 (12.9–15.0)	13.0 (11.9–14.2)	7.7 (6.7–8.9)	6.9 (5.8–8.1)
	25–29	21.1 (19.6–22.7)	19.3 (17.7–21.0)	8.2 (7.3–9.2)	7.5 (6.6–8.6)
	30 or more	25.8 (23.5–28.3)	25.7 (23.4–28.4)	10.4 (8.5–12.6)	10.1 (8.6–11.9)
Presence of chronic disease * ** ^++^	No	5.4 (4.5–6.6)	4.3 (3.5–5.4)	2.8 (2.2–3.6)	3.6 (2.8–4.7)
	Yes	22.8 (21.9–23.8)	23.2 (22.1–24.3)	11.8 (10.8–12.8)	10.8 (9.9–11.7)
Other non-psychotropic drugs * ^+^ ** ^++^	No ^†^	7.5 (6.5–8.6)	5.8 (4.9–6.8)	3.5 (2.8–4.3)	2.8 (2.3–3.4)
	Yes ^†^	23.0 (22.1–24.0)	25.0 (23.8–26.2)	12.3 (11.3–13.4)	13.2 (12.1–14.5)
Medical consultation * ^+^ ** ^++^	No ^†^	107 (9.9–11.6)	12.8 (11.9–13.7)	4.2 (3.6–4.8)	5.3 (4.6–6.0)
	Yes	28.8 (27.5–30.1)	28.7 (27.0–30.4)	17.4 (15.9–19.1)	16.6 (14.9–18.5)
Self-assessment of health status * ^+^ ** ^++^	Very good/Good ^†^	7.9 (7.3–8.7)	7.9 (7.2–87)	3.6 (3.0–4.1)	3.9 (3.3–4.5)
	Fair/Poor/Very poor	34.1 (32.6–35.6)	37.0 (34.9–38.5)	20.6 (18.9–22.4)	20.2 (18.3–22.2)

Statistically significant differences (*p* < 0.05) in women: * SNHS 2006; ** SNHS 2012; **^†^** SNHS 2006–SNHS 2012; Statistically significant differences (*p* < 0.05) in men: **^+^** SNHS 2006; **^++^** SNHS 2012; **^ǂ^** SNHS 2006–SNHS 2012.

**Table 3:** Multivariate models for associations between psychotropic drugs use and sociodemographic variables, lifestyle, health profile and healthcare resources. Spanish National Health Surveys 2006 and 2012.

Variables		Women		Men	
		SNHS 2006AOR (95% CI)	SNHS 2012 AOR (95% CI)	SNHS 2006AOR (95% CI)	SNHS 2012 AOR (95% CI)
Age	25–44	1	1	1	1
	45–64	1.91 (1.62–2.26)	1.89 (1.55–2.30)	1.02 (0.77–1.35)	1.07 (0.80–1.45)
	65 or more	1.96 (1.59–2.42)	1.85 (1.43–2.40)	0.67 (0.46–0.97)	0.55 (0.36–0.83)
Nationality	Immigrants	1	1	1	1
	Spanish	1.51 (1.09–2.10)	1.69 (1.15–2.49)	2.89 (1.55–5.40)	N.S.
Marital status	Married	1	1	1	1
	Not Married	N.S.	1.37 (1.20–1.58)	1.27 (1.04–1.57)	1.28 (1.04–1.58)
Occupational status	Employed	1	1	1	1
	Unemployed	0.93 (0.71–1.21)	1.60 (1.24–2.07)	1.77 (1.14–2.73)	1.10 (0.78–1.54)
	Inactive	1.19 (1.01–1.42)	1.88 (1.51–2.33)	2.96 (2.15–4.00)	2.85 (2.02–4.00)
Monthly income	More than 1800 €	1	1	1	1
	901–1800 €	1.30 (1.08–1.55)	N.S.	N.S.	N.S.
	900 €	1.40 (1.15–1.71)	N.S.	N.S.	N.S.
Smoking habit	Not smoker	1	1	1	1
	Smoker	1.36 (1.16–1.60)	1.39 (1.16–1.68)	1.51 (1.22–1.87)	N.S.
Alcohol consumption	Yes	1	1	1	1
	No	1.16 (1.02–1.33)	1.29 (1.12–1.50)	1.59 (1.31–1.94)	1.61 (1.32–1.96)
Presence of chronic disease	No	1	1	1	1
	Yes	1.84 (1.45–2.33)	1.78 (1.37–2.32)	1.81 (1.32–2.49)	1.47 (1.08–2.00)
Other non-psychotropic drugs	No	1	1	1	1
	Yes	1.46 (1.20–1.77)	3.00 (2.48–3.64)	N.S.	N.S.
Medical consultation	No	1	1	1	1
	Yes	2.07 (1.82–2.37)	1.50 (1.31–1.71)	2.32 (1.81–3.44)	1.83 (1.49–2.25)
Self-assessment of health status	Very good/Good	1	1	1	1
	Fair/Poor/Very poor	3.63 (3.15–4.18)	3.60 (3.12–4.17)	3.84 (3.07–4.80)	3.27 (2.62–4.07)

N.S.: non-significant association.
